# Regular swimming exercise improves metabolic syndrome risk factors: a quasi-experimental study

**DOI:** 10.1186/s13102-021-00254-8

**Published:** 2021-03-08

**Authors:** Jamal Shaker Omar, Nidal Jaradat, Mohammad Qadoumi, Abdel Naser Qadoumi

**Affiliations:** 1 Department of Physical Education, An-Najah National University, Nablus, 00970 Palestine; 2 Department of Pharmacy, Faculty of Medicine and Health Sciences, An-Najah National University, Nablus, 00970 Palestine

**Keywords:** Swimming sessions, Metabolic risk factors, Body fat percent, Palestine

## Abstract

**Background:**

In the past few decades, swimming became one of the most important physical activities within the health system and is considered a practical nonpharmacological approach to managing of type 2 diabetes (T2DM), hyperlipidemia, hypertension (HTN), and obesity. The current study aimed to assess the effect of long-term swimming sessions on glycemic and lipidemic parameters, hemodynamic responses, body fat percent, and body mass index for patients with metabolic risk factors from Palestine.

**Methods:**

Forty participants from both genders with T2DM and HTN (aged 52.4 ± 5.5 yrs) agreed to participate in this quasi-experimental study and were divided into two groups. The first group included the participants who performed long-term swimming sessions and the second group served as the control. The first group exercised for 2 h, 3 times/week in 29–33 °C swimming pool temperature for 16 weeks. Simultaneously, the control group did not participate in any exercise and advised them to keep on with their everyday lifestyle. All the obtained metabolic syndrome risk factors data were analyzed using a two-way ANOVA analysis of variance (2*2) which was applied to determine the differences according group, time, and interaction.

**Results:**

The results showed that there were statistically significant differences at *p* < 0.05 in the variables of Total Cholesterol (TC), High Density of Lipoprotein (HDL), Low Density of Lipoprotein (LDL), Triglycerides (TG), Blood Glucose (BG), Systolic Blood Pressure (SBP), Diastolic Blood Pressure (DBP), Body Mass Index (BMI), and body fat percent according to group, time, and interaction for the experimental group.

**Conclusions:**

The findings of the current study suggested that the regular 16 weeks of swimming sessions could be considered nonpharmacological approaches in managing T2DM and HTN.

## Background

Diabetes mellitus (DM) is a chronic metabolic disorder characterized by long-lasting hyperglycemia, which mainly occurs due to disturbances in insulin action, insulin secretion, or both [1]. Long-lasting hyperglycemia has been associated with harmful damages, dysfunction, and failure of different body organs like blood vessels, heart, nerves, kidneys, and the eyes’ retina, among others [2]. According to the World Health Organization (WHO), the number of diabetic patients has been quadruplicated in the last four decades [3]. Unfortunately, the prevalence of DM among the Palestinian population (East Jerusalem, Gaza, and West Bank) is about two to three times higher than in the rest of the world [4].

Hypertension (HTN) is another chronic metabolic disorder, characterized by the elevation of systolic and/or diastolic blood pressure, which can be associated with lethal complications such as heart failure and stroke [5]. The WHO estimated that about 1.13 billion of the world population reported HTN. Moreover, two-thirds of people living in the middle- and low-income countries have HTN, as reported in the same study. Despite the severity of HTN, which can cause premature death worldwide, about 20% of patients with this disease keep it under control [6].

Another metabolic disease that is threatening humanity is hyperlipidemia, in which lipids, fibrous plaques, calcium, and cholesterol can accumulate on the walls of the blood vessels. Lipid deposits can build up and result in narrowing and hardening of the arteries. Consequently, tissues and organs would not receive enough blood to function correctly. If arteries that supply the heart with blood are affected, this accumulation can narrow the blood vessel and reduce blood flow and oxygen to the heart’s muscle, which can cause angina pectoris [7]. Simultaneously, the complete blockage of the artery can cause myocardial infarction, which is commonly known as a heart attack. Besides, if the fatty matters build up in the brain arteries, that can lead to stroke if a blood clot blocks blood flow in the cervical circulation [8].

Due to DM’s harmful complications on blood vessels, HTN may occur in many diabetic patients, especially those suffering from uncontrolled blood glucose levels [9]. Therefore, it has been demonstrated that there are substantial overlaps between DM and HTN in the disease’s mechanisms and etiology [5]. HTN is documented in 2 out of 3 patients with T2DM, and its development co-occurs with the development of hyperglycemia. This association underlies many pathophysiological mechanisms, including the excitatory effect of hyperglycemia on the renin-angiotensin-aldosterone system, the stimulatory effect of hyperinsulinemia on the sympathetic nervous system, smooth muscle growth, insulin resistance in the nitric oxide pathway, the retentions of sodium and fluid. In patients with T2DM and HTN usually increases the risk of many cardiovascular diseases. According to recent guidelines, blood pressure lower than 140/85 mmHg is a reasonable therapeutic goal in patients with T2DM, and patients with controlled diabetes have the same cardiovascular risk as patients with hypertension but without diabetes [10]. T2DM and HTN tend to occur in the same patients who may also have other disorders, such as high body mass index, high-fat percentages, high plasma concentrations of insulin, and triglycerides (TG) and low serum concentrations of high-density lipoproteins (HDL) and others [11].

Accordingly, these chronic diseases must be carefully treated, and the related medical conditions require appropriate management because pathological and mental health problems may increase the risk of developing many major public health diseases, which may contribute to premature death. Lifestyle modification and glucose- and blood pressure-lowering medications are provided to keep glucose and atrial blood pressure levels close to normal [12].

One of the essential points for improving lifestyle is the continuous practice of physical exercises that significantly improve blood circulation, metabolic processes, and other physiological functions [13].

Swimming is one of the safest and gratifying methods of aerobic physical exercise [14]. In the traditional form of swimming, people can burn many calories, improve their musculoskeletal system and other physiological functions with minimal adverse effects on joints, heart, and other organs, especially in older people when swimming is conducted at a moderate or low speed [15]. Besides, moderate-intensity aerobic exercises are promising lifestyle interventions to improve patients’ cognitive function with mild cognitive impairment who have a heightened risk of developing dementia [16]. Moreover, regular aerobic exercises positively affect symptoms of attention deficit hyperactivity disorder, anxiety, depression, and other mental health disorders [17, 18].

Many scientific data described the effects of swimming and other types of aerobic physical fitness on diabetes, cardiovascular, and respiratory systems responses, and their effects on the serum lipid profile, body fat percentage, and muscle mass in subjects with T2DM [19–23].

To the best of our knowledge, investigations that describe the effects of long-term swimming sessions among patients suffering from T2DM and HTN as comorbid in Palestine are missing from the literature. Therefore, more information to evaluate the effects of regular long-term swimming procedures on blood glucose level, blood lipid profile, blood pressure, body mass index, and body fat percent in T2DM and HTN patients is required.

Therefore, this study investigates the outcomes of 16 weeks of swimming sessions on blood pressure levels, glycemic responses, lipid profiles, body mass index, and body fat percent in patients with T2DM and HTN compared with a control group suffering from these disorders without swimming sessions.

## Methods

### Participants

Forty patients (twenty women and twenty men with age 52.35 ± 5.5 yrs) diagnosed with T2DM and HTN from the employees of An-Najah National University were included in the current study. The study was conducted on a purposive sample consisting of two groups (experimental and control (of 20 patients each. Both groups included patients of both genders evenly split between males and females. The men (*n* = 20) were divided equally into two groups, experimental and control, and for women. The first group included the participants who performed long-term swimming sessions and the second group served as the control. The control group did not participate in any exercise and advised them to keep on with their everyday lifestyle.

The experimental group underwent swimming as a recreational and sports program. The intensity of the swimming was moderate and below average and did not require high effort. Each session began with a 20 min warm-up, then the participants conducted some aerobic exercises for 10 min, free-swimming for 85 min, and cool-down exercises for 5 min. While in the control group, they did not participate in any sports program throughout conducting this study, and all the participants in the current study are athletically inactive and have average skill level in swimming.

The study participants were instructed not to change their medications, diet, water intake, behavior, and sleep-rest patterns. Patients were also asked not to perform any other type of physical exercise during this experiment. T2DM and HTN medications had to be stable during the swimming sessions for all the participants.

Patients with a history of chronic pulmonary, stroke, and peripheral artery diseases also participants suffering from nephropathy, infections, peripheral ulcers, or problems with glycemic control were excluded from the current study. The participants were assigned for a 120 min swimming session, three times weekly for 16 weeks in the pool temperature of 29 to 33 °C. Clarification of the benefits, possible risks, and adverse effects of long-term swimming sessions of participating in this study were established before the participants signing the informed consent forms. The biomedical tests were taken before starting the current study and repeated after 16 weeks. The participants could stop the swimming sessions anytime they experienced an uncomfortable feeling.

### Procedures

The instructions of swimming sessions were given to the participants during 16 weeks of this study by qualified physical education researchers. Glucose levels were measured using a glucometer (OneTouch Ultra®, USA), while blood pressure was measured utilizing a mercury sphygmomanometer (Diamond BPMR120 Deluxe Conventional Mercurial Type BP Instrument, India). Moreover, the researchers calculated the body mass index and fat mass percent before and after finishing the current study. The capillary glycaemia and blood pressure measurements were taken before and after 10 min of each swimming session. All participants provided the researchers with their biochemical laboratory analysis data for TG, TC, LDL, and HDL before starting and after finishing the current experiment. However, these biochemical analyses were performed in accordance with the standard biochemical procedures and protocols. The biochemical analysis was carried out in a certified medical laboratory (Al-Wafaa Medical Laboratory, Palestine) by certified and trained laboratory technologists with experience in the use of automated analyzers. For every automated machine, daily quality assurance checks were conducted in accordance with the manufacturer’s recommendations.

### Ethical considerations

The study protocols, aims, and the informed consent forms were approved by the Institutional Review Board (IRB) at An-Najah National University (IRB archived number 3.7.2019). The study was conducted in accordance with the requirements of the declarations of Helsinki. A written informed consent obtained from all the participants before they entered in current study. Participants were not offered any incentives and they were able to withdraw from the study at any time. Data obtained was kept confidential.

### Statistical analyses

Means and standard deviations were utilized as descriptive statistics. Two-way ANOVA analysis of variance (2*2) was applied to determine the differences according to group, time, and interaction. The effect size was determined by using partial eta squared. No abnormal distributions were noticed in pretests according to Shapiro- Wilk results for both groups. Data were analyzed by using Statistical Package for the Social Sciences (SPSS) version 20 (IBM SPSS® software, USA) program, and the level of significance was fixed at *p* < 0.05.

## Results

As depicted in Table 1, the two-way ANNOVA results revealed that there were significant differences at *p* < 0.05 on all of the studied variables according to group, time, and interaction. However, in the case of group (experimental vs. control), in favor of the experimental group, there were significant differences at *p* < 0.05 on the posttest of the TC, HDL, LDL, TG, BMI, body fat percent, BG, SBP and DBP variables. On the contrary, no significant differences were found at *p* < 0.05 on the pretests of all variables between the experimental and control groups. Concerning the comparison between pre- and post-tests, the findings of the present study showed that there were statistically significant differences at *p* < 0.05 in all of the variables (TC, HDL, LDL, TG, BMI, body fat percent, SBP and DBP) in favor of posttests in the experimental group. Regarding the control group, no significant differences were found at *p* < 0.05 between the pre- and post-tests of all variables. In the case of interaction (group* time), there was statistically significant interaction at *p* < 0.05 for all of studied variables in favor of the post tests for the experimental group.

**Table 1:** Summary of two-way ANOVA results for the differences in lipidemia, blood glucose, hypertension, BMI, and fat percent according to group (Experimental vs Control), time (Pre vs Post), and interaction

Variables (units)	Group	M ± SD		Variation			Effect size (Partial Eta^**2**^)
		Pre	Post	Group	Time	Interaction	
TC (mg/dl)	Experimental	225.95 ± 20.10	173.35 ± 13.60*	0.000*	0.000*	0.000*	0.437
	Control	231.35 ± 19.97	232.55 ± 20.69				0.327
							0.350
HDL (mg/dl)	Experimental	33.35 ± 3.08	40.55 ± 2.70*	0.000*	0.000*	0.000*	0.211
	Control	33.90 ± 3.02	34.10 ± 2.88				0.296
							0.273
LDL (mg/dl)	Experimental	157.49 ± 21.31	105.71 ± 14.62*	0.000*	0.000*	0.000*	0.398
	Control	161.80 ± 19.71	162.80 ± 21.04				0.311
							0.328
TG (mg/dl)	Experimental	175.55 ± 13.06	135.45 ± 1096*	0.000*	0.000*	0.000*	0.557
							0.488
	Control	178.25 ± 7.95	178.50 ± 9.16				0.494
BMI (kg/m^2^)	Experimental	29.05 ± 1.67	26.19 ± 1.84*	0.002*	0.000*	0.000*	0.115
	Control	28.84 ± 1.59	28.80 ± 1.74				0.158
							0.151
Fat (%)	Experimental	26.89 ± 2.70	21.91 ± 2.38*	0.001*	0.000*	0.000*	0.132
	Control	26.33 ± 2.34	26.16 ± 2.25				0.229
							0.206
BG (mg/dl)	Experimental	207.55 ± 37.34	140.80 ± 19.05	0.002*	0.000*	0.000*	0.120
	Control	199.20 ± 27.12	188.35 ± 21.80				0.348
							0.217
SBP (mmHg)	Experimental	157.95 ± 12.56	134.10 ± 13.62*	0.000*	0.000*	0.000*	0.155
	Control	157.50 ± 11.33	153.80 ± 7.83				0.273
							0.167
DBP (mmHg)	Experimental	98.65 ± 3.91	87.55 ± 4.89*	0.001*	0.000*	0.000*	0.147
	Control	98.35 ± 6.50	96.50 ± 5.68				0.279
							0.165

Note values are: *M ± SD* Mean ± Standard Deviation, *TC* Total Cholesterol, *HDL* High Density of Lipoprotein, *LDL* Low Density of Lipoprotein, *TG* Triglycerides, *BMI* Body Mass Index, *BG* Blood Glucose, *SBP* Systolic Blood Pressure, *DBP* Diastolic Blood Pressure; *Significant differences at *p* < 0.05.

## Discussion

Regular physical exercises are associated with a decrease in mortality and the risk of developing various cardiovascular diseases. Physical exercises have also been found to slow aging, reduce physiological dysfunctions, and decrease DM complications by improving endurance, metabolism, muscle strength, circulation, and decreasing body fat mass [24, 25]. Besides, physically active individuals have higher insulin sensitivity, lower blood pressure, and a more favorable plasma lipoprotein profile [26]. However, to the best of the author’s knowledge, no previous data documented the effect of regular long-term swimming program on patients with chronic diseases like T2DM and HTN from the West Bank area of Palestine.

In the current study, the statistical analysis results between groups (experimental vs. control) showed that the TC, HDL, LDL, TG, BMI, body fat percent, BG, SBP and DBP variables on the posttest were significantly improved (*p* < 0.05) with effect size of partial eta squared were 43.7, 21.1, 39.8, 55.7, 11.5, 13.2, 12, 15.5, and 14.7%. While, no significant difference was found at *p* < 0.05 on the pretest of all studied variables compared to the control group.

Moreover, comparing the pre- and post-tests results, the findings of this study showed that there were statistically significant differences (*p* < 0.05) in all conducted variables (TC, HDL, LDL, TG, BMI, body fat percent, SBP and DBP) in favor of posttests in the experimental group, as the effects size of partial eta squared were 32.7, 29.6, 31.1, 48.8, 15.8, 22.9, 34.8, 27.3 and 27.9%, respectively. Whereas, no significant differences were found at *p* < 0.05 between pre- and post-tests of the studied variables in the control group.

In addition, in the case of interaction (group* time), there was statistically significant interaction (*p* < 0.05) for all of studied variables. However, the value of HDL in the post test for patients (experimental group) was significantly the greatest compared to these values of pretest (for patients in both groups) and the post test for patients of the control group, as the effects size of partial eta squared was (27.3%). Furthermore, the values of posttest for the variables (TC, LDL, TG, BMI, body fat percent, BG, SBP and DBP) among patients (experimental group) were significantly the lowest in comparison with these values of pretest for patients in both groups and in the post test for patients (control group), as the effects size of partial eta squared were 35, 32.8, 49.4, 15.1, 20.6, 21.7, 16.7 and 16.5%, respectively.

These results indicate that the participating in swimming sessions regularly has positive impact on the biochemical variables for the experimental group according to TC, HDL, LDL, TG, BG, BMI, and body fat percent tests.

It is known that physical exercises decrease blood glucose levels by increasing the sensitivity to insulin in the exercised muscle and enhancing muscle contraction-induced glucose uptake in these muscles. The mechanisms include increased hexokinase activity, increased glycogen synthase activity, decreased release and enhanced clearance of free fatty acids, increased post-receptor insulin signaling, GLUT4 mRNA and protein, and enhanced influx of glucose to the muscles due to enhanced muscle capillarization and blood flow. Also, the physical exercises enhanced the ability of the muscles to burn fat to a greater extent instead of glycogen. This is mediated by activation of a number of enzymes in the skeletal muscles that are necessary for lipid metabolism which affect positively in decreasing TG, TC and LDL and increasing the levels of HDL [27, 28].

In addition, regular training contributes into the enhancement of glucose uptake into skeletal muscles and could activate hormones like adiponectin. The higher circulating levels of adiponectin is beneficial during exercise and favors the oxidation of fat and glucose uptake into muscles. The decrease in fat and blood pressure could also be critical elements of decreasing T2DM complications [29].

In the experimental group of the current study, we noticed that the body fat percent and BMI significantly decreased as verified by the difference in results before and after the change within the group. However, the control group showed no significant difference. In fact, physical exercises can increase energy consumption and enhance lipolysis, thereby reducing the body fat mass and BMI [27].

The improvements in blood lipid profiles for experimental group lead to improvements in the blood pressure parameters while did not change significantly in the control group. In contrast, the lack of exercise causes disorder in the lipoprotein metabolism in the blood to result in an increase in the density of TC and LDL in the blood, and decrease in the HDL density which can cause various cardiovascular disorders. In fact, although the blood pressure-lowering effect of physical exercises is considered to be multifactorial, but seems to be independent of weight loss and energy expenditure. The mechanisms include neurohumoral, vascular and structural adaptation. The antihypertensive effect is believed to be mediated via reduced sympathetically induced vasoconstriction in the trained state, and decreased catecholamine levels [30].

These observations are in agreement with previously published studies which concluded that participating in aerobic exercises can improve the metabolic risk factors significantly, as well as the associated reductions in the levels of TC, LDL, TG, BG, SBP, DBP, BMI, and body fat percent and elevated the HDL level [19–21, 31–34].

Predominantly, recreational swimming exercise is based on aerobic metabolism because both lipids and carbohydrates are the primary sources of energy. For that, aerobic exercises have a positive effect on the metabolism of these substances [21]. Several published studies demonstrated a positive relationship between aerobic aquatic exercise and the right physiological and psychological conditions. These studies showed that regular physical exercises could reduce musculoskeletal disorders, many metabolic risk factors, anxiety, insomnia, depression, and stress [35–38]. Briefly, these results emphasize that practicing swimming regularly by patients suffering from Type II DM and HTN has a significant positive effect on TC, HDL, LDL, TG, BG, SBP, DBP, BMI, and body fat percentage compared to those who never participated in swimming sessions. In addition, regular swimming exercises improved metabolism rates, life style, boosting mood also increased the burning a lot of calories for these patients.

In conclusion, we recommended that T2DM, and HTN patients should regularly follow different types of training, including swimming, to have optimal health benefits [39].

### Limitations

Considerable difficulty was to recruit patients that were free from other complicating disorders and disabling conditions like the patients who have a history of chronic pulmonary diseases, stroke, peripheral artery diseases, and nephropathy. Moreover, the patients who have severe infections, peripheral ulcers, or have problems with glycemic control, which are more common in patients with the combination of T2DM and HTN, were excluded from the current study. Therefore, the sample size of the participants was small as planned by researchers. Furthermore, the current study was planned to be carried out for 12 months. Unfortunately, due to the COVID-19 pandemic, which started in our country in May 2020, we stopped the current study to protect the participants and researchers from this lethal infectious disease. A future study required to test all the metabolic risk factor markers, maximal oxygen uptake (VO2), and heart rate monitoring parameters for a more extended with a larger sample size.

## Conclusions

Sixteen weeks of regular swimming sessions resulted in significant reductions (*p* < 0.05) between pre and post-tests in blood glucose levels, lipids profiles, BMI, body fat percent, and arterial blood pressure readings for individuals with T2DM and HTN. No significant differences were found at *p* < 0.05 between pre and post-tests for all studied variables in the control group for both genders. Therefore, this kind of exercise can a useful therapeutic tool for patients with T2DM, HTN, hyperlipidemia, obesity, and overweight. This is a clinically crucial finding since a continuous swimming program can be highly recommended for individuals with HTN and T2DM. Besides, it can be useful for individuals with obesity, overweight, and hyperlipidemia.

## Data Availability

The datasets generated during and analyzed during the current study are not publicly available due to confidential information about the participants but are available from the corresponding author on reasonable request.

## Abbreviations

T2DM: Type 2 Diabetes
HTN: Hypertension
DM: Diabetes mellitus
WHO: World Health Organization
TG: Triglycerides
SPSS: Statistical Package for the Social Sciences
BG: Blood Glucose
SBP: Systolic Blood Pressure
DBP: Diastolic Blood Pressure
TC: Total Cholesterol
HDL: High Density of Lipoprotein
LDL: Low Density of Lipoprotein
BMI: Body Mass Index
